# Severe intellectual disability, omphalocele, hypospadia and high blood pressure associated to a deletion at 2q22.1q22.3: case report

**DOI:** 10.1186/1755-8166-5-30

**Published:** 2012-06-11

**Authors:** Milene Vianna Mulatinho, Cassio Luiz de Carvalho Serao, Fernanda Scalco, David Hardekopf, Sona Pekova, Kristin Mrasek, Thomas Liehr, Anja Weise, Nagesh Rao, Juan Clinton Llerena

**Affiliations:** 1 Instituto Fernandes Figueira, IFF/FIOCRUZ, Departamento de Genética Médica, Av. Rui Barbosa, 716. Flamengo, Rio de Janeiro, RJ 22250-020, Brazil; 2 Faculdade de Ciências Médicas, Hospital Universitário Pedro Ernesto, Universidade do Estado do Rio de Janeiro, UERJ, Rio de Janeiro, RJ, Brazil; 3 Laboratório de Erros Inatos do Metabolismo, Departamento de Bioquímica, Instituto de Quimica, Universidade Federal do Rio de Janeiro, UFRJ, Rio de Janeiro, RJ, Brazil; 4 Chambon Laboratory for Molecular Diagnostics (member of the Synlab Czech laboratory group), Prague, Czech Republic; 5 Jena University Hospital, Friedrich Schiller University, Institute of Human Genetics, Kollegiengasse 10, D-07743 Jena, Germany; 6 Department of Pathology and Lab Medicine, The David Geffen School of Medicine at UCLA, Los Angeles, CA, USA

**Keywords:** Array-comparative genomic hybridization (aCGH), Fluorescence in situ hybridization (FISH), 2q22 deletion syndrome, Birth defects, Hypospadia, omphalocele, Severe mental retardation, Essential hypertension, High blood pressure

## Abstract

**Background:**

Recently, array-comparative genomic hybridization (aCGH) platforms have significantly improved the resolution of chromosomal analysis allowing the identification of genomic copy number gains and losses smaller than 5 Mb. Here we report on a young man with unexplained severe mental retardation, autism spectrum disorder, congenital malformations comprising hypospadia and omphalocele, and episodes of high blood pressure. An ~ 6 Mb interstitial deletion that includes the causative genes is identified by oligonucleotide-based aCGH.

**Results:**

Our index case exhibited a *de novo* chromosomal abnormality at 2q22 [del(2)(q22.1q22.3)dn] which was not visible at the 550 haploid band level. The deleted region includes eight genes: *HNMT, SPOPL, NXPH2, LOC64702, LRP1B, KYNU, ARHGAP15* and *GTDC1*.

**Discussion:**

aCGH revealed an ~ 6 Mb deletion in 2q22.1 to 2q22.3 in an as-yet unique clinical case associated with intellectual disability, congenital malformations and autism spectrum disorder. Interestingly, the deletion is co-localized with a fragile site (FRA2K), which could be involved in the formation of this chromosomal aberration. Further studies are needed to determine if deletions of 2q22.1 to 2q22.3 define a new microdeletion syndrome.

## Introduction

Intellectual disability (ID) or Mental Retardation (MR) affects a large number of individuals, and was recently estimated to have a prevalence of 1% in the general population [1]. Chromosomal abnormalities are causative factors in 4% to 34.1% of cases, detected mainly by G-band-based banding studies [2,3]. The advent of array-comparative genomic hybridization (aCGH) has increased the detection rate by an additional 15–20% [4], generally by identifying submicroscopic chromosomal abnormalities. This progress has enabled a refined association of chromosomal aberrations and potentially underlying disease-causing genes, leading to better karyotype/genotype-phenotype correlations, and more qualified genetic counseling for families [5-8].

Genes associated with ID/MR can be found distributed throughout the human genome. According to the OMIM database [9] six genetic syndromes have been assigned to chromosomal region 2q22 to 2q23, including, Mowat-Wilson Syndrome (MWS) (MIM:235730); Nemaline Myopathy 2 (MIM:256030); Meier-Gorlin Syndrome 2 (MIM:613800); Susceptibility to Asthma (MIM:600807); Idiopathic Generalized Epilepsy 9 (MIM:607682); and, Hypogonadism, Alopecia, Diabetes Mellitus, Mental Retardation and Extrapyramidal Signs syndrome (MIM:241080). MWS is the best known disease in 2q22 ~ q23, presenting multiple congenital anomalies including Hirschsprung disease (HD) (MIM:142623) and MR. It has been recently associated with truncating mutations and/or heterozygous deletions of the *ZEB2* homeobox gene (*ZFHX1B*) [10-13].

Here we present a Brazilian patient carrying a hitherto unreported ~ 6 Mb microdeletion in 2q22.1 to 2q22.3 upstream and outside the *ZEB2* region [14]. The patient’s phenotype comprises severe autism spectrum disorder, associated to ID/MR, and congenital malformations, such as, omphalocele and hypospadia with cryptorchidism. Episodes of essential hypertension were an important feature in adolescence and were controlled with specific anti-hypertensive agents.

## Case presentation

The patient is the only son of a young non-consanguineous couple, without any familial history. He was born at term by cesarean section; weight 3.950 g (>P50^th^ percentile); length 51 cm (50^th^ percentile). The patient had his first genetic evaluation at 4 years of age, being referred due to global developmental delay, lack of speech, an omphalocele (corrected by surgery), and balanic hypospadia with bilateral cryptorchidism. At physical examination he presented a coarse face with deep-set eyes, thick eyebrows, protruding tongue, small teeth, pointed chin, bulbous nose, wide spaced and hypoplastic nipples, scoliosis, corrected balanic hypospadia with a flattened gland, bilateral clinodactyly of the fifth finger, non-specific dermatoglyphic pattern, global developmental delay and behavioral disorder. A neurological evaluation through the Childhood Autism Rating Scale (CARS) protocol revealed a score of 44.5, compatible with an autism spectrum disorder.

Clinical follow-up proceeded on different occasions, and a series of hypertension episodes were detected when the patient was 17 years old, ranging from 150x100 mm Hg to 140x80 mm Hg. His body mass index (BMI) was 40 kg/m^2^, without any echocardiogram or electrocardiogram disturbance. Complete blood count, cranial cerebral tomography, thyroid hormones and biochemical evaluation were all normal, except for high triglycerides (289 mg/dl [normal values: 50–200 mg/dl]). He was treated with an angiotensin-converting enzyme inhibitor (captopril 25 mg daily) and dietetic measures, losing more than 40 kg in 2 years.

Recently, a new clinical evaluation was performed. He is currently 23 years old and presents in good physical health, with a BMI of 27.5 kg/m^2^ and normalization of his blood pressure without the use of antihypertensive drugs. He still suffers from a severe behavioral disorder, occasional tantrums, stereotyped movements of his trunk and repetitive whistling. Such clinical signs have improved dramatically with the use of antipsychotic drugs. Differential diagnoses such as Smith-Magenis, Simpson-Golabi-Behmel and Beckwith-Wiedemann syndromes have been ruled.

## Results

**Figure 1:**
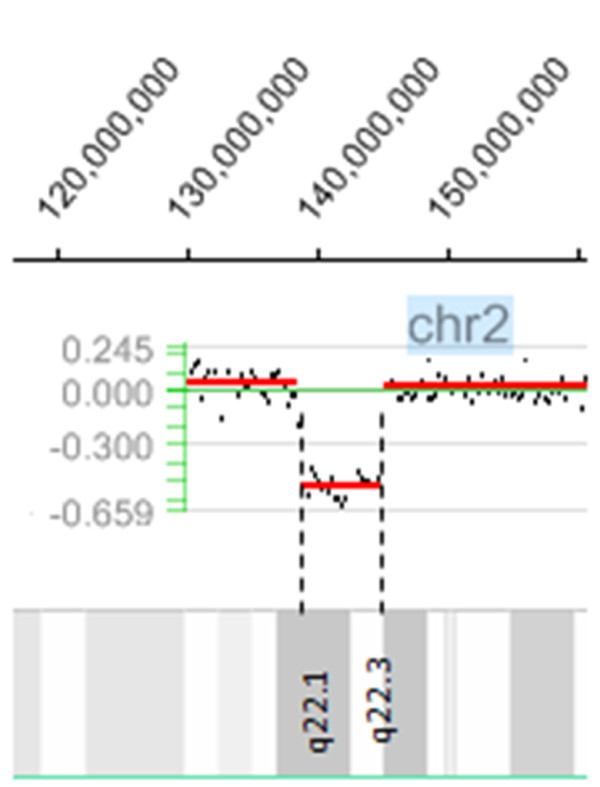
**NimbleGen whole-genome oligonucleotide aCGH profile for chromosome 2q.** The ideogram (grey bars) depicts 2q22.1 to 2q22.3 genomic regions with the cytogenetic bands on the long arm of chromosome 2 (not on scale). The ~ 6 Mb interstitial deletion interval at 2q22.1–q22.3 [hg19, chr2: 138,750,000–144,750,000 bp] is indicated by a red horizontal line below zero and the two black dotted vertical lines.

**Figure 2:**
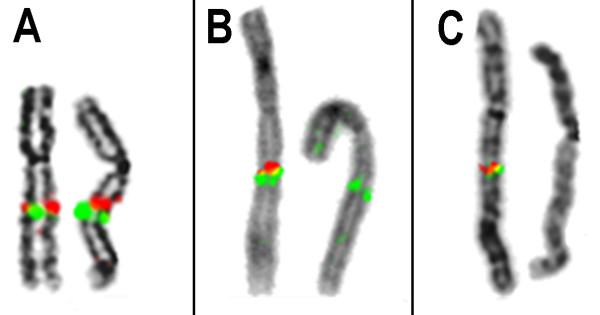
**Three pairs of chromosome 2 are shown to illustrate the FISH results obtained with the BAC probes. a)** RP11–587 K7 in 2q23.3 (green) and RP11–112 N16 in 2q22.1 (red) were located outside the deleted region. **b)** RP11–64O2 in 2q22.3 (green) is located outside the deleted region, while RP11–731 F1 in 2q22.1 (red) is within the affected region. **c)** RP11–137 J9 (green) and RP11–164E7 (red) both are missing on the derivative chromosome 2, indicating deletions in 2q22.1.

In the current case, G-banding and subtelomeric screening as well as molecular testing for the *FMR1* and *FMR2* genes were all normal (data not shown). aCGH identified an interstitial deletion of 6 Mb in the long arm of chromosome from 2q22.1 to 2q22.3, spanning positions 138,750,000 to 144,750,000 (Figure 1) and comprising eight genes (Table 1) [14]. The molecular cytogenetic karyotype according to ISCN 2009 was designated as: arr 2q22.1q22.3(138,750,000–144,750,000)x1. Seven out of 10 FISH probes used in the 2q22.1 band confirmed the deletion (Table 2). Eight to fourteen metaphase spreads were evaluated (Figure 2). The absence of xanthurenic acid in the patient’s urine showed that the metabolic pathway of tryptophan was not altered.

**Table 1:** **Genes within the 2q22.1 to 2q22.3 deleted region according to OMIM**[9]

***gene symbol***	***name***	***OMIM #***	***description***	***function***
***HNMT***	NM_006895	605238	Homo sapiens histamine N-methyltransferase (HNMT), transcript variant 1.	It metabolizes Histamine in mammals. This gene encodes the first enzyme, which is found in the cytosol and uses S-adenosyl-L-methionine as the methyl donor. This variant (1) represents the longest transcript and it encodes the longest protein (isoform 1).
***SPOPL***	NM_001001664		Homo sapiens speckle-type POZ protein-like (SPOPL), mRNA	-
***NXPH2***	NM_007226	604635	Homo sapiens neurexophilin 2 (NXPH2), m RNA.	-
***LOC647012***	NR_033658		Homo sapiens YY1 transcription factor pseudogene (LOC647012), non-coding RNA.	-
***LRP1B***	NM_018557	608766	LOW DENSITY LIPOPROTEIN RECEPTOR-RELATED PROTEIN 1B	LRP1B belongs to the low density lipoprotein (LDL) receptor gene family. These receptors play a wide variety of roles in normal cell function and development due to their interactions with multiple ligands.
***KYNU***	NM_001032998; NM_003937	605197	Homo sapiens kynureninase (KYNU)	Kynureninase is a pyridoxal-5'-phosphate (pyridoxal-P) dependent enzyme that catalyzes the cleavage of L-kynurenine and L-3-hydroxykynurenine into anthranilic and 3-hydroxyanthranilic acids, respectively. Kynureninase is involved in the biosynthesis of NAD cofactors from tryptophan through the kynurenine pathway. Alternative splicing results in multiple transcript variants.
***ARHGAP15***	NM_018460	610578	Homo sapiens Rho GTPase activating protein 15 (ARHGAP15), mRNA	RHO GTPases (see ARHA; MIM 165390) regulate diverse biologic processes, and their activity is regulated by RHO GTPase-activating proteins (GAPs), such as ARHGAP15
***GTDC1***	NM_018460; NM_024659; NM_001006636	61065	Homo sapiens glycosyltransferase-like domain containing 1 (GTDC1)	GTDC1 is ubiquitous expressed at relatively high levels in lung, spleen, testis, and peripheral blood leukocytes, suggesting that it may have biochemical functions in these organs.

**Table 2:** **FISH probes used inside the 2q22.1-2q22.3 region to confirm the array data**[15,16]

***Locus***	***signals***	***BACs***	***Accession number***	***Start position (bp)***	***End position (bp)***
**2q22.1**	2x	RP11-112 N16	AC010873	137,567,308	137,747,509
**2q22.1**	del	RP11-731 F1	AC069394.6	138,791,256	138,964,607
**2q22.1**	del	RP11-597P14	AC097523	138,954,985	139,129,617
**2q22.1**	del	RP11-231E19	AC092620.2	139,299,060	139,450,096
**2q22.1**	del	RP11-137 J9	AC092837	139,462,901	139,629,396
**2q22.1**	del	RP11-432O12	AC023468	139,608,867	139,779,582
**2q22.1**	del	RP11-15D9	AC109345	139,736,474	139,903,043
**2q22.1**	del	RP11-164E7	AC108036	141,266,879	141,423,790
**2q22.3**	2x	RP11-64O2	AQ237761 AQ237759	145,181,324	145,355,222
**2q23.3**	2x	RP11-58 K7	AQ201454 AQ201457	153,589,449	153,743,069

Human Genome Assembly Build 37 (hg19) shows that the region 2q22.1 to 2q22.3 is covered by small copy number variations, inversions (structural variations) and InDels, but no sequence gaps.

## Discussion

A 23-year-old patient with ID/MR, autism, essential hypertension, and congenital malformations including an omphalocele and hypospadias with cryptorchidism is reported. He carries an ~6 Mb de novo microdeletion at 2q22.1–22.3 identified by an oligonucleotide aCGH panel [arr 2q22.1q22.3(138,750,000–144,750,000)x1 dn] according to Human Genome Assembly Build 37 (hg19).

Table 3 summarizes the clinical and genomic data from our index case and four patients described in the Decipher database with overlapping deletion intervals (138,750,000 to 144,750,000 bp) [17]. ID/MR is a common clinical feature, but none of the other listed cases presented with congenital malformations such as those found in our patient. Besides ID/MR, two patients show other common clinical features: strabismus and thick eyebrows (Patient 2566); and strabismus, bulbous nasal tip, and hypoplastic/inverted/absent nipples (Patient 1607). Interestingly, Patient 1607 has a complex karyotype involving chromosomes 2, 3 and 5 [18]. None of the individuals listed in Table 3 shared the exact breakpoints at 2q22.1–q22.3 as observed in our patient [17].

**Table 3:** Present case and four patients listed at Decipher with similar deletions, ranging from 2q22.1 to 2q22.3, are shown

***Patient***	***Cytogenetic deletion***	***sex***	***Interval start-end (bp, hg19)***	***Interval (Mb)***	***RefSeq Gene***	***Phenotype***
**This report**	2q22.1q22.3	Male	138,750,000–144,750,000	6	*HNMT, SPOPL, NXPH2, LOC647012, LRP1B, KYNU, ARHGAP15, GTDC1*	Omphalocele, cryptorchidism, hypospadia. ID/MR, deep-set eyes, strabismus, thick eyebrow, protruding tongue, small teeth, pointed chin, bulbous nose, wide spaced nipples, hypoplastic nipples, bilateral clinodactyly of fifth finger, non-specific dermatoglyphic patterns, scoliosis, global developmental delay and behavioral disorder. Autism spectrum disorder.
**1607**	2q22.1q22.3	Female	139,813,180–145,063,389	5,25	*HNMT, SPOPL, NXPH2, LRP1B, KYNU, ARHGAP15, GTDC1*	ID/MR, strabismus, bulbous nasal tip, hypoplastic/inverted/absent nipples.
**2566**	2q22.2q22.3	Female	143,635,233–147,935,002	4,30	*LRP1B, KYNU, ARHGAP15, GTDC1*	ID/MR, strabismus, thick eyebrows.
**250662**	2q22.1q22.3	Male	141,232,786–147,935,002	6,70	*HNMT, SPOPL, NXPH2, LRP1B, KYNU, ARHGAP15, GTDC1*	−
**251811**	2q22.2q22.3	Female	143,715,235–146,369,069	2,65	*LRP1B, KYNU, ARHGAP15, GTDC1*	−

LEGEND: important data from five individuals with overlapping intervals are shown. The RefSeq genes and the phenotype columns list only the genes and the clinical findings shared with our patient. Full information of gene contents and phenotype from the Decipher patients is seen at Decipher website [17].

A child with MWS presenting with delayed psychomotor development, hypotonia, a variety of dysmorphic features, genitourinary anomalies and a severe course of HD has been described with a deletion at 2q22.2 to 2q22.3 [143,468,147–147,106,860] [19]. This 3.6 Mb aberration included *ZEB2* and three other genes not currently associated with disease-*KYNU, ARHGAP15* and *GTDC1*-all encoding for proteins involved in ubiquitous and non-specific pathways [20-22]. This deletion segment overlaps with our case in an ~ 1.2 Mb [143,468,147–144,750,000] comprising *KYNU, ARHGAP15* and *GTDC1* (Table 1). The authors speculate that those genes could play a crucial role in the process of tissue regeneration [19]. While many candidate genes have been studied to investigate their role in birth defects such as omphalocele and hypospadias/cryptorchidism [23-25], the clinical observations in our patient suggests the assignment of such malformations to the genes in the region 2q22.2–2q22.3.

Particularly interesting seems to be the function of the gene *KYNU*. It has previously been mentioned as possibly participating in a three-gene interaction influencing hypospadia, cryptorchidism and/or omphalocele [19]. However, a polymorphism in *KYNU* has also been linked to essential hypertension in a group of Han Chinese [26]. This feature has been investigated by studies that show the influence of *KYNU* as a candidate for hypertension in spontaneously hypertensive rats [27,28]. *KYNU* encodes kynureninase, a vitamin B6-dependent enzyme involved in the kynurenine pathway for the biosynthesis of NAD cofactors from tryptophan, and its deficiency has been associated with abnormal tryptophan metabolism (MIM:605197) [22]. A massive urinary excretion of xanturenic acid known as hydroxykynureninuria or xanturenic aciduria (MIM:236800) can be detected in cases of kynureninase deficiency, due to defects in the kynurenine pathway [22,29]. Based on the literature, an investigation was done to detect the presence of xanthurenic acid in our patient’s urine to examine the function of this gene. This biochemical study showed a normal level of xanthurenic acid, indicating that the tryptophan pathway is not affected and with a likely normal gene function. This result is in accordance with the low rank of 91.4% in the Decipher database, indicating that this gene is more likely to not exhibit haploinsufficiency [17]. Finally, genes and/or susceptibility loci on the long arm of chromosome 2 have been recently linked to blood pressure and hypertension by genome-wide association studies, such as *STK39* at 2q24.3 [30]; *PMS1* and *MSTN*, both at 2q32.2 [31]; DS2S2382 and DS2S338 at 2q35–q37 [32].

Within the region 2q22.1–2q22.3, no functions have yet been assigned to *SPOPL, NXPH2* or *LOC647012. HNMT* is the only gene currently associated with human disease (Asthma) [9]. Our patient, however, has not shown any episodes of asthma. Special attention should be given to *LRP1B*, which is a newly identified member of the LDL receptor family. It was originally described as a putative tumor suppressor in lung cancer cells, but its expression profile in several regions of the adult human brain such as cortex, hippocampus and cerebellum suggests it may have additional functions in the central nervous system [33-35]. Its interaction with the β-amyloid precursor protein could protect against the pathogenesis of Alzheimer’s disease [33]. Expression of this gene has also been reported in the thyroid and salivary gland [34].

*LRP1B* is a very large human gene (1,9 Mb), located at 2q22.1 close to the fragile site, FRA2K, at 2q22.3 [36]. Many large genes residing within unstable chromosomal regions are highly evolutionarily conserved, and in general are not traditional mutational targets; however, genomic alterations can occur due to fragile site instability and contribute to diseases, including a variety of cancers [37]. Furthermore, there are important potential linkages between such genomic alterations and neurological development or neurodegeneration, for e.g. *CNTNAP2* (2,3 Mb) localized within FRA7I at 7q35 found disrupted in a family with Gilles de la Tourette syndrome [38], and *PARK2* (1.3 Mb) mutated in autosomal recessive juvenile Parkinson disease and located in the active center of FRA6E at 6q26 [39].

Fragile sites are understood to be specific loci that preferentially exhibit gaps and breaks in metaphase chromosomes following partial inhibition of DNA synthesis, and their break-prone areas are almost equally distributed along chromosomes [36,40]. Human chromosome 2 has the highest number of fragile sites, with twenty one break-prone regions spaced at an average distance of 11.52 Mb [36]. The deletion studied in this report is placed at 2q22.1q22.3, overlapping FRA2K (2q22.3) and preceeded by FRA2F at 2q21.3. This is in line with the fact that regions of chromosomal instability at or near fragile sites are hot-spots for deletions and other alterations [41].

The fragile site neighboring 2q22.1q22.3 may have facilitated the chromosomal aberration in our patient including the entire *LRP1B* gene region. Moreover, a haploinsufficiency rank of 13.8% was recently established for this gene, just above the 0–10% range indicating a high likelihood of exhibiting haploinsufficiency [17]. If *LRP1B* is haploinsufficient in our patient, and considering its biological function within the central nervous system, it is tempting to speculate on the participation of this gene in the patient’s observed cognitive impairment. In addition, the presence of SNP variant rs2890652 (142,676,401) in *LRP1B* has been associated with BMI by genome wide association analysis [42]. While it is clear that correlation with potentially functional variants does not prove that these variants are causal, they can provide initial clues into which genes might be prioritized in further studies [42]. Consequently, *LRP1B* should be the subject of further studies to assign its relationship with BMI. The clinical management for hypertension and BMI in our patient at the age of 17 years included the use of an angiotensin-converting enzyme inhibitor and a vigilant diet.

## Conclusion

Here we describe a patient presenting severe ID/MR, autism spectrum disorder, dysmorphism and congenital malformations, with episodes of high blood pressure associated with high levels of BMI. A whole-genome aCGH screening revealed an approximate 6 Mb *de novo* deletion, and a review of the literature provides indications of a new contiguous gene syndrome located in 2q22.1 to 2q22.3.

## Methods

Peripheral blood chromosome analysis at the 550 G-band level was performed applying standard cytogenetic procedures. Molecular testing for *FMR1* and *FMR2* genes was performed [43,44]; and the subtelomeric ToTel Vysion panel of probes (Abbott–Vysis) was also done. DNA from the patient was isolated from lymphocytes according to standard protocols and was subjected to aCGH analysis [Human Whole-Genome CGH; NimbleGen Systems, Madison, WI] to evaluate the presence of pathogenic copy number changes. The platform contained 385.000 oligonucleotides at a median spacing of 6 kb. The data was analyzed with the NimbleGen SignalMap v.1.9 software. Fluorescence in situ hybridization (FISH) using standard protocols with the following BAC clones as probes were used to confirm the deletion: RP11-112 N16, RP11-731 F1, RP11-597P14, RP11-231E19, RP11-137 J9, RP11-432O12, RP11-15D9, RP11-164E7, RP11-64O2, RP11-58 K7 (Table 2) [15,16].

Urinary organic acids were analyzed to evaluate the metabolic pathway of tryptophan once the *KYNU* gene was found to be deleted inside the 2q22 region (Table 1). This biochemical analysis was performed by high-resolution gas chromatography coupled to mass spectrometry (Agilent 5975 C, HP-5).

The family consented to participate in the study, which was approved by the Brazilian Ethical Committee Board.

## Consent

Written informed consent was obtained from the parents of the patient for publication of this case report and any accompanying images. A copy of the written consent is available for review by the Editor-in-Chief of this journal.

## Abbreviations

CARS: Childhood Autism Rating Scale; FISH: Fluorescence in situ hybridization; ID/MR: Intellectual disability/Mental retardation; ISCN: International system for human cytogenetic nomenclature; aCGH: array-comparative genomic hybridization; BMI: Body Mass Index; MWS: Mowatt Wilson Syndrome; HD: Hirschprung disease.

## Competing interest

The authors declare that they have no competing interests.

## Authors’ contributions

MVM drafted the manuscript, performed the cytogenetic analysis and the FISH subtelomeric screening, the molecular analysis for *FMR1* and *FMR2*, and analyzed and interpreted the aCGH data. CLCS and JCLJr carried out clinical examination and evaluation of the patient. FS performed the biochemical analysis. DH and SP isolated and provided the BAC probes. KM performed the BAC-FISH relevant confirmation. AW, TL, NR and JCLJ coordinated the study. All authors improved and approved the manuscript.
